# DNA Studies: Latest Spectroscopic and Structural Approaches

**DOI:** 10.3390/mi12091094

**Published:** 2021-09-11

**Authors:** Monica Marini, Francesca Legittimo, Bruno Torre, Marco Allione, Tania Limongi, Luciano Scaltrito, Candido Fabrizio Pirri, Enzo di Fabrizio

**Affiliations:** 1 Dipartimento di Scienza Applicata e Tecnologia (DISAT), Politecnico di Torino, Corso Duca degli Abruzzi 24, 10129 Torino, Italy; francesca.legittimo@polito.it (F.L.); bruno.torre@polito.it (B.T.); tania.limongi@polito.it (T.L.); luciano.scaltrito@polito.it (L.S.); fabrizio.pirri@polito.it (C.F.P.); enzo.difabrizio@polito.it (E.d.F.); 2 Istituto Italiano di Tecnologia (IIT), Via Livorno 60, 10144 Torino, Italy; marco.allione@iit.it

**Keywords:** nucleic acids, microfabrication, electron microscopy, atomic force microscopy, Raman spectroscopy

## Abstract

This review looks at the different approaches, techniques, and materials devoted to DNA studies. In the past few decades, DNA nanotechnology, micro-fabrication, imaging, and spectroscopies have been tailored and combined for a broad range of medical-oriented applications. The continuous advancements in miniaturization of the devices, as well as the continuous need to study biological material structures and interactions, down to single molecules, have increase the interdisciplinarity of emerging technologies. In the following paragraphs, we will focus on recent sensing approaches, with a particular effort attributed to cutting-edge techniques for structural and mechanical studies of nucleic acids.

## 1. Introduction

In this review, we report on novel approaches and innovative technologies devoted to the study and characterization of deoxyribonucleic acid (DNA), one of the most important molecules in life science and medicine. We will focus on the more recent advances, as well as the combination of different materials, technologies, and fields of research, ranging from physics to molecular biology. These advances have led to the development of novel methods and original techniques to solve biological questions, and to unveil technical challenges and limits.

## 2. Overview of Nucleic Acids

### 2.1. DNA as Genetic Material

**Figure 1:**
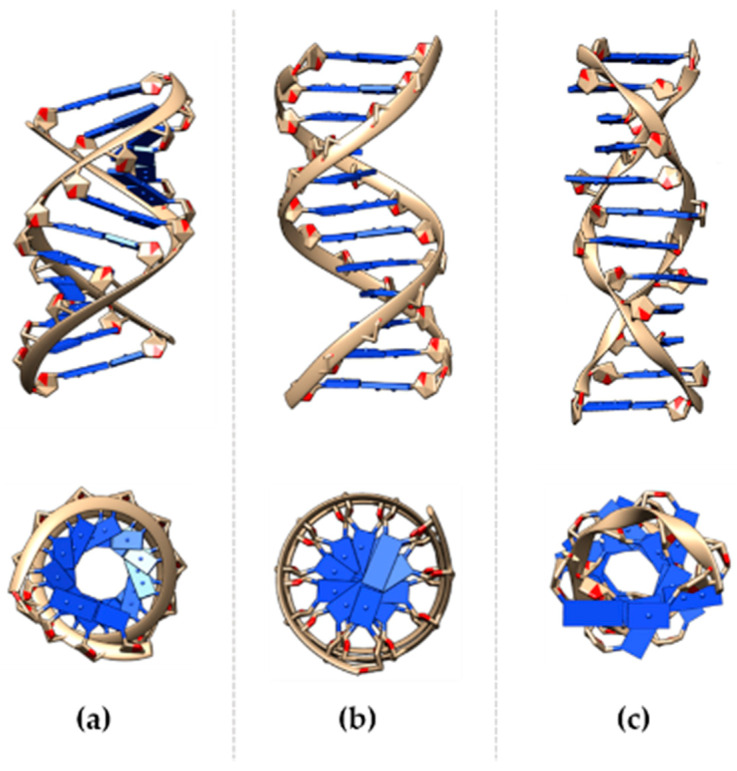
DNA helix in the (**a**) A-, (**b**) B-, and (**c**) Z-form lateral and upper view. A- and B- DNA structures were built and viewed using the program Chimera [3] for the arbitrary sequence ATCGATCGATCG; Z-form refers to the dodecamer TCGCGCGCGCGCG [4].

Nucleic acids are a class of macromolecules that store genetic information. They comprise ribonucleic acid (RNA) and deoxyribonucleic acid (DNA), which play different roles using slightly different chemical compositions. DNA is a double-stranded (ds) molecule composed of nucleotides as monomers. Each nucleotide has three components: Nitrogen base, a phosphate group, and the deoxyribose sugar. The bases are divided into pyrimidine (T, thymine, and C, cytosine) or a purine base (A, adenine, and G, guanine), while the sugar and phosphate form the DNA backbone. The double helix has two anti-parallel strands, which are kept in place by hydrogen bonds between complementary base pairs. Hydrogen bond disruption causes hemihelices separation, resulting in single strand (ss) DNA. There are three main families of DNA helices: A-, B-, and Z-DNA forms (Figure 1) [1,2].

Right-handed A- or B-DNA, and left-handed Z-helices show different structural characteristics, and thus functional relevance. Despite the B-DNA form, it is mostly known to be the “ideal” DNA structure, other conformations are commonly present in physiological conditions. The double helix assumes conformations in response to environmental stresses, such as hydration [5,6] and the ionic environment [7,8,9]. Helical conformation variance is also induced by the sequence of the bases [10], e.g., the A-form can be induced by purine-based fragments, while the B-DNA is mostly favored by mixed bases. The transition between DNA-forms is caused by chemicals interactions [11], such as with ruthenium and spermidine, as well as by methylation events [12], where a methyl group is added to the fifth carbon of the cytosine residue. The recognition and interaction of the double-strand with proteins during relevant cellular processes can be maximized, optimized, and produced by local variations to the B-form of the nucleic acid, as reported for a large number of DNA-binding proteins [13,14,15].

A-form is the prominent conformation in low humidity (<75%) conditions. Each helix has 11 bases per turn, 2.6 Å rise per base pair (bp), a diameter of approximately 23 Å, and the deoxyribose pucker are 3′-endo. The base pair plane is tilted at ~19° to the helix axis, and contributes to the formation of the major (deep and narrow) and the minor (wide) groove. This DNA form is more rigid, compared to B-form, and is commonly associated with heteroduplexes (RNA-DNA) and low hydration conditions; the sequence-inducing conformational transition has been demonstrated.

B-DNA is considered the “canonical” helix conformation and is the favored form in a high humidity (>75%) and mixed bases sequences, with 10.5 bp per turn, 20 Å diameter, 3.4 Å rise per base pair, and the sugar pucker is C2′-endo. B-DNA helix axis is almost perpendicular to the plane of the base, and has two grooves, known as the “major” (wider) and “minor” (narrower) groove.

Z-form of DNA has been identified in the late 70s and differs substantially from the canonical B-form. It is a highly-ordered structure with 12 bases per turn, a narrower diameter (18 Å) compared to the other forms of DNA, has a wider axial rise (45 Å). Notably, the major groove is almost non-existent, while the minor groove is narrow. Tracts alternating dinucleotide repeating purine-pyrimidine or pyrimidine-purine (APP) sequences are known to adopt the left-handed conformation [16,17], and are over-represented in higher eukaryotes. In fact, GpC repeats, also named “CpG islands”, show a strong attitude to crystallizing as a Z-structure [18].

Polynucleotides sequences can arrange in non-canonical structures, such as triplex, G-quadruplex, and i-motifs [19]. Triplex motifs were discovered in 1957 and consist of sequences of purines and pyrimidines, which are held in place by Watson-Crick base pairing, while a third sequence of pyrimidine or purine interacts with them through Hoogsteen base pairing [20]. DNA triplex can be intra- and inter-molecular. Inter-molecular triplexes are formed by binding an exogenously-applied oligonucleotide to a target duplex sequence. Naturally occurring intramolecular triplexes (H-DNA), formed at endogenous mirror repeat sequences, present exploitable features that permit site-specific alteration of the genome. G-quadruplex (G4) were first described in 1910 [21] and then detailed in the 1960s [22]. G4 are formed by a tetrad of guanine bases, especially in G-rich sequences [23]. The G-core structure is stabilized by the presence of monovalent cations, such as K^+^ and held together by Hoogsteen hydrogen bonds [24]. Multiple G4s can combine in different topologies based on tetrads directions and sequence lengths. DNA sequences, rich in cytosine, present so-called intercalated motifs (i-motifs). Quadruplex structures were hemi-protonated cytosines (C–C^+^) and can be stabilized by H-bonds [25,26]. These non-canonical structures participate in fundamental cell processes, such as replication or transcription, and can be used as therapeutic targets in response to disease [27,28]. DNA can be collected from a variety of samples, ranging from tissues to serum by commercial kits and known protocols. Shorter arbitrary sequences (i.e., oligonucleotides) can be easily addressed, designed, modified, synthesized, and ordered from dedicated companies. The architectural peculiarities of the macromolecule, such as autonomous self-assembly through precise base-pairing, conformation manipulation, sequence predictability, innate thermodynamic stability [29], and biocompatibility offer the chance for DNA to become a fundamental constructive material in nanoscience and nanotechnology.

### 2.2. Nucleic Acids as Constructive Material: DNA Nanotechnology

DNA nanotechnology has shown increasing potential in its application in several areas, spanning from life sciences to plasmonic and biophysics. Recently, we have seen a rise in structures with programmed features devoted to the predesigned assembly of, e.g., nanoparticles [30] for different purposes, such as biosensing, imaging, and detection [31,32,33]. In recent years, various dynamic plasmonic DNA structures, based on the controlled structural reconfiguration after the exposure to the environment were realized, e.g., reversible and light-responsive DNA-locks [34], DNA-metamolecules to manipulate chirality [35], and plasmon rulers to monitor gold/silver nanoparticles distance and separation [36]. DNA-based structures and their configurations also appear in this review in the following paragraphs given their historical and technological relevance. In the proceeding paragraphs, we address specific and dedicated works, and briefly introduce the basic concepts for discussion. The fabrication of nanometer-scale objects is based on two approaches: Top-down and bottom-up. The top-down approach starts from large structures and reduces to nano-sized materials, but then rapidly faces increasing difficulties in reaching a molecular scale size. On the contrary, the bottom-up approach uses objects at the nano- or sub-nanometer scale (e.g., atoms or molecules) to build up and self-assemble nanostructures. Molecular self-assembly results in numerous periodic features with symmetric, ordered, programmable, and well-organized structures. In this context, DNA has become an outstanding material for the synthesis of nanostructures and nanomaterials. In the last 40 years, several constructs exploiting base-pairing interactions between synthetic nucleic acids have been assembled, e.g., artificial DNA-junctions since the early 80s [37,38], rigid triangles [39], cubes [40], nanowires [41,42], and several complex shapes [43]. Linear oligonucleotides have also been used to assemble branched structures called “dendrimers” with several exposed DNA ramifications available for functionalization [44]. DNA origami was introduced by Rothemund in 2006 and has quickly become a landmark technique [45]. Based on this approach, a long single-stranded polynucleotide, named “scaffold strand”, runs back and forward to fold into the designed nanostructure. It is held in place with the help of hundreds of shorter complementary oligonucleotides, known as the “staple strands”. Through a precise software-based design (CaDNAno [46,47], SARSE [48], CanDO [49,50]) and the proper thermal treatments, it was possible to create planar objects (smiles, squares, triangles, circles, rectangles, stars [50,51]), as well as three-dimensional structures (cubes, cylinders, gears [33,34,52]). In all cases, the DNA objects are addressed. These nanostructures can be rigid or dynamic, such as in the case of DNA walkers [53], cylinders [54], disks [49,55], and cages [56].

Complementary approaches, based on smart substrates design were developed to organize and process genetic material, as described in Section 3.

## 3. Smart Substrates for Nucleic Acids Characterization

In the following paragraphs, we provide an overview of the innovative ideas that can exploit nucleic acid characterization. The aim is to highlight new experimental approaches that are under constant development, aside from the most established approaches. Therefore, this review does not comprehensively address all techniques that investigate molecular life. The reported approaches are summed up in Table 1 to guide the reader through their continuous development.

### 3.1. Superhydrophobic Devices

Superhydrophobic surfaces (SHS) are substrates by which the water contact angle is above 150°. These systems have been known for a long time, since many natural biological systems have displayed such effects (the leaves of some plants, e.g., lotus, or insects wings are well-known examples) [144]. In the laboratory, this effect has been replicated several times and has been studied extensively, both for fundamental reasons and for its application in many fields. The superhydrophobicity of a surface typically emerges as an interplay of materials and morphology. In the laboratory, the most straightforward way to replicate this behavior is to create a hydrophobic surface, and possesses several pillars or tips coming out of the surface, in either a regular or random arrangement, with its only requirement being that the fraction of the surface, which is covered by pillars (or any other elevated structure) must remain within a specific range. In this case, a water drop deposited on such a surface finds a more energetically favorable environment by minimizing the contact surface with the sample by lying suspended on top of the surface tips or pillars. On the macroscopic scale, the global effect appears as a drop lying on the surface with a very small contact area and a corresponding large contact angle, which is around 170° in some cases [145].

There is broad range of possible techniques and available materials that can be used to realize such specimens. A common option is to exploit the multi-decades experience, built in the microelectronics and MEMS industry, in order to machine silicon at micro/nanoscale to realize silicon patterned surfaces by deep reactive ion etching. This well-established technology enables patterns of almost any arbitrary shape to be machined down to almost any arbitrary depth. In fact, different shapes and regular patterns can be defined on the surface of a pristine substrate via one of the different standard lithographic techniques (optical by laser scanning or by transfer from a mask, or electronic), and then the pattern can be engraved in silicon down to a certain depth with greater control and reproducibility. One of the advantages of this type of technology is that it can also produce samples with pillars on very thin silicon wafers or membranes, which can also be locally perforated in spaces between adjacent pillars. Such samples are extremely interesting as they are compatible with electron microscopy analyses of suspended material. A slightly different alternative is to realize the structured surface by directly exploiting the resist used in the lithographic process. This leaves the possibility of using different substrate materials with respect to silicon, providing an opportunity to obtain, for example, completely transparent devices.

Several polymeric materials and resists are available for this purpose, commercially available, and present advantages, such as physical characteristics, transparency, flexibility, and low costs in large-scale production. However, a more extensive understanding of silicon fabrication makes it the preferred choice in many cases as it allows for more flexibility in design. For example, if it is necessary to cover the top part of the pillars only with gold, this production method combines such particular surface coatings within the fabrication process quite easily. Superhydrophobicity can be improved, when required, by additional surface hydrophobic coating. Since these surfaces have structures with potentially high aspect ratios, conformal coating techniques, like vapor-phase or plasma-assisted depositions are preferable to other more directional techniques, e.g., evaporation in high vacuum. Some common examples of such coatings, include plasma-assisted deposition of a Teflon-like polymer using a C4F8 precursor. Another example is a vapor-phase deposition of self-assembled monolayer coatings of molecules, which have an adhesion functional group attached to a hydrophobic tail, like per-fluorodecyltrichlorosilane (FDTS). These conformal deposition techniques can uniformly coat any surface of the sample at any orientation.

The application of superhydrophobicity and patterned devices to the biological field is broad and still growing. Superhydrophobic coatings with antibacterial, antiviral, and antimicrobial properties have been reported [146,147], as well as nanostructured devices for cells and platelets adhesion or repellence [148], cell culture, tissue engineering, drug delivery [149], biosensing [150,151], and proteins analysis [152].

In this review, we focus on the application of superhydrophobic devices for structural studies purposes, such as high-resolution direct DNA imaging (Section 6.2) and spectroscopic analysis devoted to the DNA conformational investigation (Section 5.1.1).

### 3.2. Microfluidics: From DNA Processing to Structural Analysis

Microfluidics is the study of the science and technology that process small volumes of fluids (10^−9^ to 10^−18^ L) constrained into micrometric/nanometric geometrical features of a chip [153]. A microfluidic chip contains microchannels molded or etched from a substrate where the fluids are directed, compartmentalized, and manipulated. At the microscale, the fluid flows laminarly; while, the main mechanism for mixing is diffusion. Diffusion allows for the finer control of the mixing conditions, and this, combined with the possibility of operating with smaller volumes of samples and reagents, makes microfluidics an attractive field for biosensing and molecular analysis. Microfluidics greatly benefits from the advances of the past decades in nanofabrication and miniaturization technologies, as well as plasmonic and DNA nanotechnologies. There is a wide range of materials that can be chosen for the chip fabrication. Moreover, there are several fabrication methods available, and tailored to, the final application of the chip. Detailed reviews on the manufacture of microfluidic devices are already available and addressed in [154]. In this paragraph, we address the latest applications of microfluidic devices to DNA analysis.

#### 3.2.1. DNA Microarray and Microfluidic Array Devices: Labelled-Based Approaches

In 1979, Kafatos, Weldon, and Efstratiadis described the Dot-Blot technique for the indirect evaluation of relative concentrations of nucleic acids in a mixture. This method consists of the immobilization of a plasmid DNA target of an unknown sequence on a single nitrocellulose membrane in dots of a homogenous diameter. Then, a solution of radioactively-labeled DNA probes are released onto the membrane under hybridization conditions, and the amount of hybridization events with each DNA dot is evaluated semi-quantitatively with autoradiography [57]. The use of porous membranes allows large DNA surface binding, but it was not possible to reduce the size of single dots beyond certain limits and to exactly control their shapes. However, these are the compelling requirements for the automated and large-scale analysis of hybridization signals needed for fast DNA-sequencing or genetic screening. For this reason, in the past several decades, a large variety of DNA-microarray devices were produced and thousands of known sequence nucleic acid probes could be placed onto a glass substrate, e.g., through ink-jet, light directed fabrication techniques, and in-situ synthesis. Hybridization with labeled targets produces mainly a fluorescent signal, directly proportional to the number of hybridization events. From the position of the luminescent spots, it is possible to reconstruct the sequence of the target DNA and/or the concentration of the target in solution. These devices have largely been implemented for diagnostics and Single Nucleotide Polymorphism (SNP) detection [58,59], but the diffusion-based DNA target-probe hybridization is time-consuming. The duration of the process is several hours to days, as the diffusion constant of DNA in water is extremely low (*D*_w_ = 4.9 × 10^−9^ cm^2^/s × [bp]^−0.72^, *D*_w_ = 2.1 × 10^−7^ cm^2^/s for 80 mer) [60].

In this context, the implementation of DNA microarrays into microfluidic devices led to the reduction of the solution volumes up to micro- or nano-liters, implying shorter diffusion lengths. This process, combined with a continuous target-DNA solution flow in the hybridization region, greatly reduces the hybridization time to achieve faster and more uniform hybridization. Moreover, miniaturization levels of microfluidic devices allowed multiple preparative steps to be merged, such as DNA extraction from cells, followed by polymerase chain reaction (PCR) in a single chip, giving rise to the Lab-on-a-chip (LOC) technology [61], e.g., in reference [60], a PMMA serpentine microfluidic channel is integrated into a microarray device. Hybridization occurs on the surface of discrete plugs shuttled back and forth along the channel by the circulating fluid. Using this strategy, the hybridization time drops from 18 h to approximately 8 min and the detection volume is reduced to 1 μL. Recently, Xiong et al. [62] reported on the implementation of a magnetic nanomixer in the hybridization region, which enhanced the kinetics of DNA microarrays four-fold. Huang et al. [63] also provided an intriguing device devoted to the point mutation detection of genetic variants in inherited arrhythmic diseases. Based on the Finite Element Method (FEM) simulation results, the researchers designed a microchannel with an elliptical shape and two columns of rectangular pillars in the hybridization region, which expanded the parabolic flux. The introduction of reciprocal flow increased the hybridization efficiency. Through microarrays probes, three variants of the human cardiac sodium channel alpha-subunit (SCN5A) gene were found to be implicated in arrhythmic diseases. As a proof of concept, a target DNA, wildtype (WT), and mutated (MU) sequences were collected from patients. After the hybridization between probe and target, a staining agent (SYBR green I) was added. Intercalant increases its fluorescence preferably upon the recognition of double strands and gives a slight fluorescent background in ssDNA presence. To avoid this issue, a solution of Graphene-Oxide (GO) was introduced in the system, which efficiently quenched the fluorescence of single-stranded DNA. This led to excellent discrimination between hybridized and not hybridized probes, and thus led to the detection of MU and WT exons after only 45 min, using a simple measure of the fluorescence intensity with an inverted microscope. This is a promising result as the device was able to implement several processes (including target DNA loading, buffer exchanging, washing, and staining) in a single automated platform. On the other hand, the GO quenching effect is strongly dependent on the GO sheets size and shape, which requires 3 h of ultrasonic process. This affects the reproducibility of the system and the general fruibility of the device, inducing the requirement for large-scale test for massive deployment.

#### 3.2.2. DNA Microarray and Microfluidic Array Devices: Label-Free Analysis

A label-free analysis does not require the hybridization of labels to targets or DNA probes. Among many detailed reviews on the topic, as in [66], we focus on the remarkable recent findings in the field. We will organize this paragraph into two main topics: Optical and electrochemical devices, according to the physical event on which the detection relies.

##### Optical Devices

**Figure 2:**
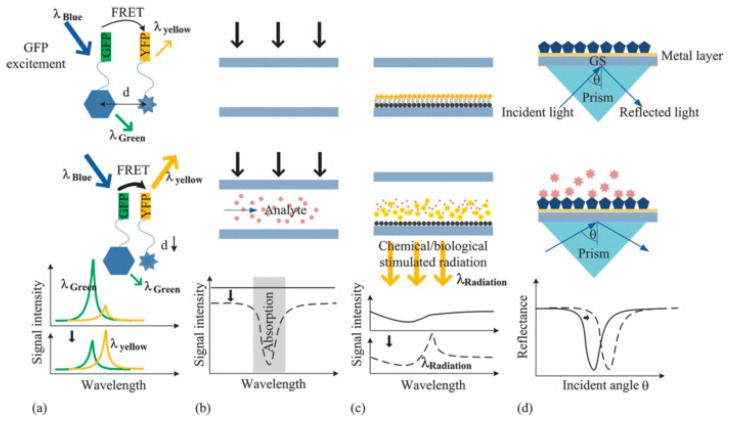
Optical methods. (**a**) Fluorescence (in this case, fluorescent resonance energy transfer/FRET), (**b**) absorbance, (**c**) luminescence, and (**d**) surface plasmon resonance (SPR)-based optical detection methods. Reprinted by J. of Biomedical Optics, 16(8), 080901 (2011) © (2011) Society of Photo-Optical Instrumentation Engineers (SPIE) [64].

Optical devices can identify hybridization events by direct methods (fluorescence, absorbance, and luminescence-based methods) and by monitoring light properties modulation, such as in the case of Surface Plasmon Resonance (SPR). The possible approaches are addressed in Figure 2 [64]. SPR biosensors rely on the detection of a refractive index change at a metal surface after the functionalization with a probe molecule. In the work by R. D’Agata et al. [65], an ultrasensitive nanoparticle-enhanced plasmonic method has been used for the detection of extremely diluted (1 aM) single nucleotide variants of Rat viral sarcoma (RAS) gene collected from plasma of colorectal cancer patients. The approach relies on the detection of cell-free (cf-DNA) and circulating tumor DNA (ct-DNA) released in the peripheral blood. The presence of ct-DNA can promote the development of new metastases [155]; since the mutation of RAS and other genes often occurs in oncologic patients, the temporal tracking of mutation of ct-DNA can be a remarkable diagnostical tool. In [65], the authors used plasmonic detection, enhanced by gold nanoparticles (Au-NPs), in order to distinguish mutated or wild type (MU or WT) RAS gene sequences directly in the plasma, without the need for preliminary genetic material extraction, purification, or amplification. The device consists of a gold chip with immobilized MU-DNA and WT-DNA probes placed in a different position. The presence of an integrated six-channels microfluidic device allowed the direct, precise, and independent spatial control of several areas of the surface at the same time. Plasma collected from patients and healthy controls are loaded on the hybridization region under continuous flow. Oligonucleotides, complementary to the part of the WT or MU-DNA probe, and functionalized with Au-NP, are placed in a region not involved in the hybridization process. This step boosts the plasmonic signal, detected through a SPR-imager apparatus. The output is converted into percentage of reflectivity of the chip, which is directly related to the amount of hybridization events. The Limit of Detection (LOD) was 2 pg/μL, which was promising for the implementation of a non-invasive approach.

#### Electrochemical Devices

In the last decade, electrochemical biosensors were widely explored given their inexpensive costs, easiness of implementation, and low LOD [66,67]. The devices based on Field-Effect Transistor (FET) are remarkable. These devices operate as intrinsic amplifiers and convert small changes in surface potential into large current variations. Therefore, they are perfect candidates for the ultralow detection sensitivity required, e.g., for cf-DNA or ct-DNA. The transconductive material used for the sensitive part of the FET, i.e., drain-to-source channels has been widely investigated [68,69]. Early FET sensors were made using traditional semiconductors (e.g., Si) and oxides (e.g., Al_2_O_3_), but were often limited in sensitivity, due to their low surface-to-bulk ratio. Graphene has been used to overcome this limit. It has unique properties for the purpose. It can be doped directly by the absorbed analytes and can be easily deposited through ink-jet printing. In several works, graphene-based bioFET showed promising results [70]. In recent work, the authors described the first gated FET obtained on commercially available Printed Circuit Board (PCB) [71]. The core board was used as the substrate, while the drain, source, gate, and reference pads were electrodes, which achieved the highest miniaturization level possible. The graphene channel was deposited between the drain and the source through a two-steps drop-cast process, and then the DNA probe was immobilized onto it. After having established the FET channel current modulation through electrical measurements inside a Faraday cage, the hybridization step was performed by easily dropping DNA solution on the top of the device. Systematic positive shifts of the channel current over the voltage gate curve were obtained with increasing complementary DNA concentrations (from 100 pM to 1 μM). The LOD of this device is still lower (1 nM) compared to more established techniques [72], given the absence of micro-fluidic integration. However, this work is the starting point for the development of Lab-on-PCB, which proposes the integration of electronics, sensing, fluidic channels, and packaging by well-established PCB protocols.

#### 3.2.3. High Resolution Melting Analysis: Structural Studies

An extensive amount of research related to DNA analysis for the detection and sensing has been reported for microfluidic devices. Fewer papers have been devoted to structural analysis. Structural variations, such as the presence or absence of methylations into a DNA sequence is of fundamental importance in gene expression. Among their biological key role, such features lend to different properties of the double helix based on their percentage within the biomolecule. A promising approach to studying such epigenetic features is the high-resolution melting analysis (HRMA). Briefly, amplified DNA is intercalated with a dye, which gives a fluorescence signal if the nucleic acid is in the double-strand form. It is quenched when the double helix is denatured by heating, and hence appears as two hemi-helices. The melting temperature (T_m_) is defined as the temperature at which 50% of the DNA strand has been denatured and is strongly related to the DNA sequence and epigenetic features [73]. At the end of the heating process, a melting curve is recorded where the fluorescence signals are related to temperature, and associated with the sequence. The comparison of the experimental results with the reference melting curve showed that it is possible to investigate the presence of nucleotide variations and methylations.

In [74], the authors integrated HRMA in an all-in-one HYPER-Melt platform, consisting of a microfluidic device with a combined optical and thermal platform. The authors analyzed the synthetic sequences of the tumor suppressor gene CDKN2A with an increasing percentage of methylation (0%, 33%, 67%, 100%). Each sample was composed of a pool of oligonucleotides where the unmethylated sequence was included as a reference. The samples were combined as follows: 0% + 33%, 0% + 67%, and 0% + 100%. The melting curves acquired showed inflection points defining the melting temperature, which increased with the methylation percentage of the sequences used. To evaluate the feasibility of this approach with real samples, in the same work, the authors used genetic materials from patients. The strong clinical potential of the platform has also been confirmed through a study of the tumor suppressor gene *NDRG4*, which has been found to be methylated in colorectal cancer. Liquid biopsies from healthy and colorectal cancer patients were collected and studied. Three out of four colorectal cancer-positive patients were positive for *NDRG4* methylation by using the HYPER-Melt platform, while only one of the four healthy volunteers tested positive. This work underlines the importance of structural differences and their consequences due to epigenetic variations in the specific case related to methylation density.

## 4. Insights into Helix Morphology: Microscopic Approaches

### 4.1. Transmission Electron Microscopy

Transmission electron microscopy (TEM) has been widely applied to biomolecule analysis. Imaging on commercial carbon grids needs molecules to be deposited, and they are usually stained to maximize the signals under the high-energy working conditions of conventional TEM. Negative and positive staining are commonly used to visualize soft materials. Positive staining relies on scattering agents interacting with the sample, such as osmium tetra oxide [156]. On the contrary, negative staining produces a layer of electron-dense stain in which the sample is embedded [157]. The particles are surrounded by agents, such as uranyl acetate and appear as a brighter area in support of imaging. Although those protocols are widely applied to macromolecules complexes, they face limitations, such as hidden inner details of the samples, dimensions, and structure alterations or damage due to dehydration and cross-linkage with stains. Moreover, the contrast achieved can be low and supports non-conductive properties below 4 nm thickness, which inevitably decreases imaging quality [158]. Although several biological complexes were visualized with this method achieving a resolution of around 20 Å, in this review we focus on DNA double helix details imaging [159]. Therefore, recent technical/protocol advances that do not rely on staining are explored. Different attempts to visualize unstained nucleic acids have been provided in the last years. The search for high resolution imaging and new applications has led to continuous improvements and development of EM techniques and related materials. Among these, it is worth citing the use of graphene as an atomically thin supporting material, as well as cryogenic electron microscopy (cryo-EM).

Recently, graphene has been used in support of biosamples, rather than conventional carbon-coated grids. DNA adsorption on graphene surfaces occurs by π-π stacking interaction [160,161], and finds its applications in biosensing [75], nanoelectronics [162], and TEM imaging. The use of graphene as a TEM support [163] was convincing for several reasons. It is an ordered and periodic structure [164], thus allows for, in some cases, image subtraction if needed. Graphene shows mechanical strength, the ability to withstand high acceleration voltages (up to 300 keV [165]), and reduces beam damage. Moreover, it is an atomic film of carbon [166], and the low scattering cross-section promotes background noise reduction [76]. Buckhout-White attempted to image DNA on graphene in 2013 by growing a layer of graphene through low-pressure chemical vapor deposition (LCV) and transferring it to a silicon membrane of a grid [77]. Graphene was then exposed to a poly-L-lysine (PLL) treatment, followed by DNA deposition, and rinsing with water. The final dry etching removed the Si membrane to make the graphene layer with the sample available for imaging. The authors recorded micrographs of unstained DNA objects, such as a tetrahedron and an 8 × 16 nm rectangle; the sizes imaged were coherent with the designed ones despite objects shrinkage and deformation and undefined edges. In 2017, Kabiri et al. imaged stained and unstained DNA nanostructures, and compared the results between the use of conventional carbon membranes and free-standing graphene films. The authors combined aberration-corrected scanning transmission electron microscopy (STEM) at 300 keV and dark-field (DF) microscopy to maximize nucleic acids contrast. The distortion of rectangular-like 2D-origami, detected after adsorption on graphene was further investigated, and the results suggested a substrate-dependent deformation. Shape edges were blurred, masked by the background pattern of graphene. Although the appealing properties of graphene films, the hydrophobic interaction with DNA remains a hurdle [76,167]. However, in the case of unstained DNA, the contrast on graphene layers is fainted by the similar composition and by the sizes and periodicity of aromatic rings presents, both in DNA and the substrate. The contrast and resolution resulting in graphene use, until now, is comparable to the one reported for common amorphous membranes representing the limit for supported samples. Moreover, at the time of writing, nucleic acids imaging attempts often rely on the use of big DNA nanostructures, thus losing the information regarding the fine details of the single helix.

A huge advancement in biological studies has been driven by Cryo-EM, which began to grow in the 1980s, and has recently has been widely applied to high molecular weight biomolecules, including complexes with nucleic acids and nanostructures. Cryo-EM does not need a reduction in the sample into crystals as in traditional X-ray crystallography. The sample is quickly frozen on a grid, cooled with liquid nitrogen, and then imaged. This approach relies on the acquisition of hundreds to thousands of images of the particles dispersed on the grid with different orientations, which is followed by extensive image processing and class averaging. The result is a map (cryoEM-map) of the sample, used to elaborate a model of the object imaged with the best resolution possible, allowing for macromolecule analysis at a sub-nanometre scale. The final reconstructed image is strictly dependent on the technical advances in microscopy, as well as the continuous evolution and improvement in data processing. Recently, DNA-based objects were reconstructed with resolutions around 10 Å [78,79], while for order proteins >100 kDa, the resolution increases to 2–3 Å range with the best result for apoferritin structure (~481 kDa) with 1.54 Å resolution [80]. Cryo-EM hugely contributes to structural biology. However, the low signal-to-noise ratio and low throughput makes the imaging and reconstruction of asymmetric and small molecules, as well as single DNA helices, extremely challenging.

### 4.2. Atomic Force Microscopy

Since its invention [168], Atomic Force Microscopy (AFM) was recognized as a very promising candidate for biomolecule investigations. The main strengths of this technique in its application to biomaterials are outlined as follows:

1. the possibility to routinely and easily access the few nanometers resolution limit (and below), breaking the Abbe limit of conventional optical microscopy [169];
2. the possibility of easily working in liquid and on insulating materials, making it one of the elected techniques for biological specimens under different physiological environments [169,170,171];
3. the probe is inherently a force sensor with tens of piconewton resolution, allowing for very gentle mechanical probing and molecular manipulation, as well as sectioning at the same time. This enables the design of a variety of experiments, including protein unfolding/unzipping, antibody/antigen recognition, or chemical recognition and bonding using functionalized tips [172,173,174];
4. sample preparation is relatively easy for its operation in physiological/in vivo applications, since atomically flat, inert substrates are commercially available, and a variety of protocols for specific functionalization of the target have been developed over years [174,175].

For these reasons, from the very first years after its invention, AFM was regarded as a disruptive advancement in the microscopy field, especially in liquid applications where other sub-diffraction techniques, such as Scanning Tunnelling Microscopy (STM) and Scanning Electron Microscopy (SEM) readily showed important limitations [176]. Beginning from the late 1980s, many technical advances triggered new applications and boosted the throughput of scientific results in biology in the following decades [170,177]. In 1988, for the first time, microfabrication was used to develop reliable cantilevers as for sensors, obtaining atomic resolution [178]. In the same year, Mayer and Amer introduced the first optical beam-based force detection system, which is the most common architecture and remains in use, representing a substantial step toward the use of this technology in everyday applications [179]. In 1989, the first commercial AFM was launched. Triggered by these advancements, Bustamante et al. [81] obtained the first reliable imaging of DNA on mica in air. The method involved the replacement of K^+^ ions by Mg^2+^ at the surface to strongly electrostatically attract the phosphate backbone of DNA. The first Imaging in tapping modes dates back to 1993 by the Hansma research group [82]. This was an important development, since dynamic modes opens up the possibility of investigating mechanical properties, while acquiring topography. Cantilever sensors were used by Bockelmann et al. [83] to perform DNA unzipping experiments to asses thermodynamic properties of the bonding. The technique was further developed and used by Gaub group in the following years [84]. More extensive reviews on this topic can be found in recent literature [180]. In recent years, two important technological advancements were developed in new studies. First, the attempt to access a higher frame rate was introduced as a prototype by T. Ando [181,182] and pursued with different strategies [183]. This provided an opportunity for different commercial instruments to image and perform force spectroscopy at a higher video rate. This meant information on events could be gathered in the few tens of milliseconds range, allowing the possibility of investigating faster dynamics in biological events. High speed AFM has been used to study the process of DNA-protein interaction [85], DNA-translocation and looping by restriction enzyme EcoP15I [86], and DNA translational freedom [87], among other biological materials [88,89].

The second advancement involves the development of the dynamic (Tapping) mode in the analysis of harmonic distortion and/or frequency shifts to extract mechanical properties, while gaining stable imaging of the molecules with the same spatial resolution. It resulted in improved resolution using a better control of tip sample interaction. A variety of techniques were performed to achieve this aim [90,91,92,93]. Recently, AFM amplitude and frequency checking has been used to resolve different structural conformations of the DNA double helix. Similarly, it was possible to correlate the effect of supercoiling on DNA flexibility and major groove recognition by triplex-forming oligonucleotides, combined with molecular dynamics. This shows the ability to bridge timescale gap accessibility using the two approaches [94].

## 5. Insights into Double Helix Structure and Analysis: Spectroscopic Approaches

In this Section, we explore recent advances in the structural characterization of DNA and their interaction with the environment limited to spectroscopic application, such as Raman and enhanced spectroscopy related techniques and mechanical evaluation through vibrometric approaches. To understand the recent advances in the fast developing and huge fluorescence community, it is possible to address reference [184], while reference [185] addresses the X-Ray technology.

### 5.1. Raman and Fourier Transform Infrared Spectroscopy

Raman spectroscopy is a vibrational spectroscopic method, which relies on the inelastic scattering of light by a sample irradiated with a laser source, due to the energy exchange between the photons and the molecular bonds vibrations in the specimen. Each spectral contribution to this scattering brings information on the energy of a specific vibrational mode of the sample, thus providing a signal bringing a huge amount of information on the chemical and molecular structure of the material analyzed. The information can be label-free, related to multiple analyte signals, or to a specific biomarker. Raman spectroscopy can provide a wide range of information when applied to the study of DNA molecules. Similar to its use in molecules, DNA spectra shows a characteristic fingerprint [95] related to feature presence or absence and their relationship to the environment. Arising out of the chemical data in DNA spectra, insights on hydrophobicity, H-bonds, conformational, and structural variations can be obtained [96]. Interactions with compounds spanning from ions to macromolecules such as proteins can also be revealed [97,98]. Recently, Raman spectroscopy and SHS were integrated to provide a comparative study of ssDNA and dsDNA, as well as the assignation of the A- and B- conformation to suspended DNA fibers [99,100]. Raman spectroscopy poses low sensitivity detection limitations as only one in approximately 10 million photons is inelastically scattered [101]. This is lower than the typical autofluorescence background of biological solutions. Among vibrational spectroscopic techniques, the so-called surface-enhanced Raman spectroscopy (SERS), tip-enhanced Raman spectroscopy (TERS), and Fourier transform infrared spectroscopy (FTIR) provide fingerprint spectra of biomedical samples, including nucleic acids. These techniques represent complementary real-time analytical methods for non-destructive and rapid DNA analysis.

#### 5.1.1. SERS

A powerful method for overcoming Raman spectroscopy hurdles and for studying the chemical structure of biological samples was discovered many decades ago. Fleischman and colleagues found that some metal surfaces and nanostructures could be the source of small spots of concentrated electromagnetic field when irradiated by light [102]. In these so-called hot spots, the enhancement of the electromagnetic field compared to the intensity of the original incoming field strength can be enormous. It can lead to a consequent huge local enhancement of the Raman scattering, which can grow so high so that it makes it possible to detect the Raman signal form even single isolated molecules. SERS consists of a development of Raman scattering and provides superior sensitivity. Through the tailored bond of dedicated functional groups it can exploit molecular structural specificity [103]. Since its discovery, SERS has inspired significant interest and led to the demonstration of many detectors, in particular, bio-detectors for a large range of applications. For the very extensive number of different implementations and applications of SERS-based detectors, one can refer to [104,105]. This work is limited to some of the most recent developments.

**Figure 3:**
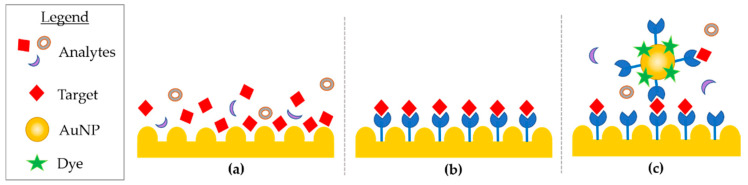
(**a**) Direct, (**b**) indirect, and (**c**) extrinsic SERS.

In detail, SERS enhancement results from the contribution of an electromagnetic and chemical phenomena [106]. The electromagnetic phenomena arises from the plasmonic nano-features (e.g surface roughness, nano-structures, and nanoparticles), the enhanced optical properties of gold (Au) and silver (Ag), which are able to produce localized electromagnetic field (EF) when the feature’s dimension is smaller than the wavelength of the interacting light. The strong confined EF of the plasmonic substrate is used to magnify the signal of analytes with low Raman scattering cross-section when they are in close proximity to the plasmonic substrate or particle [107]. The huge advancements in the design and fabrication methods of a wide range of plasmonic substrates supported the improvement of SERS analytical sensitivity. The first generation SERS substrates were comprised of two-dimensional plasmonic devices, and silver or gold spherical and anisotropic (cubic-, triangle-, rod-, and star-shaped) plasmonic nanoparticles [108,109]. SERS has been successfully used for the study of nucleic acids, such as whole genes, oligomers, and mononucleotides [110,111,112]. SERS can be divided into three methods, mostly identified as direct, indirect, and extrinsic approaches (Figure 3).

In direct SERS analysis, the sample is directly deposited on the nanostructured device and the spectra acquired. This approach is straightforward as it requires little sample preparation and allows the whole vibrational spectra of molecules to be collected, such as DNA. On the other hand, the analyte can suffer from a weak affinity to the metal surface, and from the need to acquire data from a purified biomolecule [113]. Nucleic acids adsorption onto metal nano-patterned surfaces have some hurdles. In ssDNA, the negatively charged backbone is repelled, but the exposed bases can interact with the surface despite a different affinity. In this case, adenine dominate SERS spectra as it has the major affinity. In dsDNA, base pairs are less available to the substrate, as they are hidden by the backbone [114]. Further, the use of SERS aspecific surfaces for nucleic acid analysis might not provide enough indicators for biomedical applications [115], due to their material small cross section. Accordingly, several examples of indirect SERS analyses were developed. Although this characteristic can present limits in some applications, it can be an advantage for others, such as methylation analysis. SERS spectra of peripheral blood genomic DNA, extracted from acute myeloid leukemia and from healthy cells were compared and analyzed, showing a difference in the band intensity related to 5-methylcytosine. The band was weaker in DNA from cancerous cells, suggesting hypomethylation in accordance with previous studies [116]. The significant differences in structural properties of cancerous DNA are due to the pattern of methylations, strongly affecting the DNA adsorption [117], and resulting in enhanced adenine SERS spectra. Many tunable plasmonic nanostructures sensors as nanowires, nano-stars, nano-shells, nano-spheres, nano-flowers, and three dimensional SERS hotspot matrixes were successfully used for the evaluation of DNA mutation and methylation [118,119,120,121,122]. Recently, the direct SERS detection of nucleic acids was coupled with polymerase chain reaction (PCR) to detect ct-DNA mutations by using spermine-functionalized Ag NP as a plasmonic substrate able reduce noise by minimizing the adsorption of aspecific molecules and interfering signals [113]. In the past several decades, three-dimensional nanoscale plasmonic surfaces and NP assemblies have been developed as the next generation SERS plasmonic “hot spots”, where SERS signal is maximized by the confinement of the electrical field into a few nanometer squared area. The possibility of developing precise hot-spots enhances the sensitivity at localized sites, i.e., hot spots were recently obtained through silver self-similar chains (SSC) designed nanoparticles [123]. In the research by Coluccio et al., three in-line silver nanostructures were obtained by electron lithography followed by electroless metal deposition; the smallest gap between NP was of 10 nm and represents the hot-spot, which maximizes the enhancement for short oligonucleotides. Other approaches rely on the aggregation of silver nanoparticles promoted by aluminum ions addition with the aim to obtain several hot spots, hence SERS signal of structural features of biological relevance, such as G-quadruplexes [124], i-motifs, and C-rich sequences in ssDNA [125].

Indirect SERS analysis uses molecules immobilized on the nano-patterned surfaces or particles, available for recognition of the analyte, which is brought in close proximity to the SERS surface. The spectra acquired before and after hybridization are then used to identify the analytes. In direct SERS approaches, the whole intrinsic fingerprint of the molecules is acquired. In indirect SERS methodologies research, many detection applications rely on the use of short synthetic oligonucleotides that can functionalize metals with well-established protocols. In recent years, the attention has moved to genomic dsDNA, increasing the complexity of the system, as targets are composed of thousands of bases and require PCR amplification [126,127]. PCR and asymmetric PCR [128] techniques for the detection and multiple detection of mutations [112], While, ct-DNA [129] were integrated in extrinsic SERS approach. In extrinsic SERS, a label or tag, named “Raman reporter” is introduced to provide a detection signal. The target analyte is immobilized on the support and then is exposed to a SERS probe. The probes are designed to have a large cross-section, and labelled with Raman reporters (i.e., commercial fluorescent dyes) and a molecule extremely selective towards the target. The probe provides an enhanced signal of the label, and thus target successful DNA capture. The acquired spectral features exclude matrix interference and provide information on the precise presence of a feature, but loosen intrinsic vibrational information. The selective response of extrinsic SERS is well-suited for bio-sensing purposes.

In all the cases, SERS showed unique properties in biosensing and structural investigations. Among the number of different applications, SERS potential was demonstrated in several fields, spanning from detection to medical-related advances, i.e., through the assessment of the genomic DNA cancer-related methylation pattern [122].

#### 5.1.2. TERS

TERS combines Scanning Probe Microscopy (SPM) capabilities with SERS enhancement to allow the morphological characterization of DNA sequences and provide a Raman fingerprint. It is a valuable alternative to PCR amplification or fluorescent labelling [130,131,132]. TERS relies on the amplification of the EM field localized at the tip of a plasmonic metal probe. A tailored experimental design, such as a gap-mode design with a silver tip coupled with a gold substrate, resolved a ssDNA molecule with sub-nanometer spatial resolution [130]. Lipiec et al. characterized DNA double strand breaks by TERS studying the DNA bonds sensitivity of circular plasmid DNA to ultraviolet C radiation [133]. The examination of significant bands related to P-O-H and CH2/CH3 bending modes, O-C cleavage resulted to be the main type of DNA damage. TERS assisting in successful in the nanoscale spectral study of organized DNA-based nanostructures, as ds λ-phage DNAs combed on octadecyltrichlorosilane (OTS)-modified borosilicate glass surface have been examined by using Ag/Au coated tips with an enhancement factor higher than 6 × 10^2^ and a lateral spatial resolution better than 9 nm. Whereas, the TER spectra of nucleobases is consistent with (ss) DNA, and additional modes related to the DNA backbone can be detected in the ds DNA [134].

#### 5.1.3. FTIR

Infrared spectroscopy techniques, such as FTIR have often been used as precise, conservative techniques for the study of structure and dynamics of genomic or synthetic DNA samples, including poly- and oligo-nucleotides. Since the IR spectrum of DNA is extremely conformation-sensitive, it is possible to evaluate the extent of DNA-bound water. In [135], a DNA film was achieved by drying the nucleic acids solution on a crystal and the spectrum has been recorded. DNA film dehydration modifies the B–A transition typical IR absorption spectrum. Paston and colleagues demonstrated that the volume of water that deeply interacts with DNA decreases by increasing Na^+^ ions concentration. FTIR spectroscopy assist the scientific community in understanding phenomena, such as the interaction of active substances and drugs with DNA. Batista et al. evaluated the effects of anti-neoplastic compounds administered to cells by FTIR Attenuated Total Reflectance (SR-FTIR-ATR). The cells were exposed to two cisplatin-like Pt/Pd-drugs [136], and the DNA extracted. The key pharmacological targets and the related DNA configuration changes on breast cancer cells after treatment have been characterized.

### 5.2. Vibrometer

In 2017, C. M. Domínguez at al. [137] studied the vibrational properties of gold-coated silicon microcantilevers, grafted with thin films of DNA, and constructed through the self-assembly of Sulphur tethered ssDNA oligonucleotides. The aim of this work was to study how the hydration levels (relative humidity, RH) and the water-DNA intermolecular forces affect the averaged mechanical properties of the 2D DNA film. The influence of the steric effects was also considered by varying the DNA-grafting density. Remarkably, the researchers were able to simultaneously follow the static bending and the dynamic resonance frequency of the cantilever as a function of an RH, ranging from 0% to 70%. In this way, it was possible to uncouple the effect of the inertial mass added to the resonator through grafting from the change in elastic properties due to hydration. This approach showed that, for a surface density of 3.7 × 10^13^ molecules/cm^2^, the Young’s modulus increases from ~5 GPa to ~10 Gpa when RH changes from 0% to 70%. Their results were confirmed by molecular dynamics simulations, and clearly asserted the possibility of finely-tuning ssDNA elasticity, which could be useful in many applications ranging from DNA nanotechnology to drug-delivery. It is worth noting that almost the same approach was used by the same group [138] to detect the presence of specific long DNA fragments (200–300 bp), belonging to the *blaOXA48* gene, in a lysate sample without prior purification or amplification.

## 6. Unconventional Approaches

### 6.1. Plasmonic Enantiomer Separation

Microfluidic devices with a high control level of fluid direction, position, and mixture can be also efficient tools for the futurist envisioning of bottom-up synthetic biology, which aims to reconstruct cellular phenomena in vitro [186]. On the other hand, DNA nanotechnology can provide highly sensitive and programmable structures. An efficient combination of these two technologies can boost the field of synthetic cells. An important step in this direction has been developed by K. Göpfrich et al. in [139], where the encapsulation and actuation of DNA-assembled dynamic nanostructures inside cell-sized microfluidic sections was reported for the first time. They constructed a DNA origami cross-structure, with two arms; the subunits can be locked or unlocked by a pH-sensitive DNA switch, based on a triplex motif. A gold nano-rod is attached to each origami arm to facilitate the optical detection of conformational changes of the cross structure through circular dichroism (CD) spectroscopy. However, the revolutionary contribution of this work is given by the injection of the aqueous solution containing that origami structures into a microfluidic polydimethylsiloxane (PDMS)-based droplet formation device. Cell-sized water-in-oil compartments are formed when the aqueous face is intersected by a transversal oil phase. As a result of this process, the nanostructures are encapsulated, forming plasmonic droplets, stabilized by the surfactants. Furthermore, they established a non-invasive approach for increasing the pH inside the droplets without interfering with the droplet integrity through the addition of pyridine to the oil phase of the droplet emulsion. However, it is particularly worth noting that this combination of DNA nanotechnology and droplet-based microfluidics led to an on chip filtration and separation of plasmonic enantiomers. In fact, they encapsulated a mixture of right-handed (RH) and left-handed (LH) plasmonic origami crosses with cholesterol-tagged DNA. The cholesterol-tagged DNA self-assembles into a surfactant layer, which selectively anchors to LH species, while RH ones remain in solution. The RH structures are released by breaking up the droplets. Whereas, the LH structures remain bound to the water−oil interface, achieving plasmonic enantiomer separation.

### 6.2. SHS for DNA Structural and Mechanical Studies

**Figure 4:**
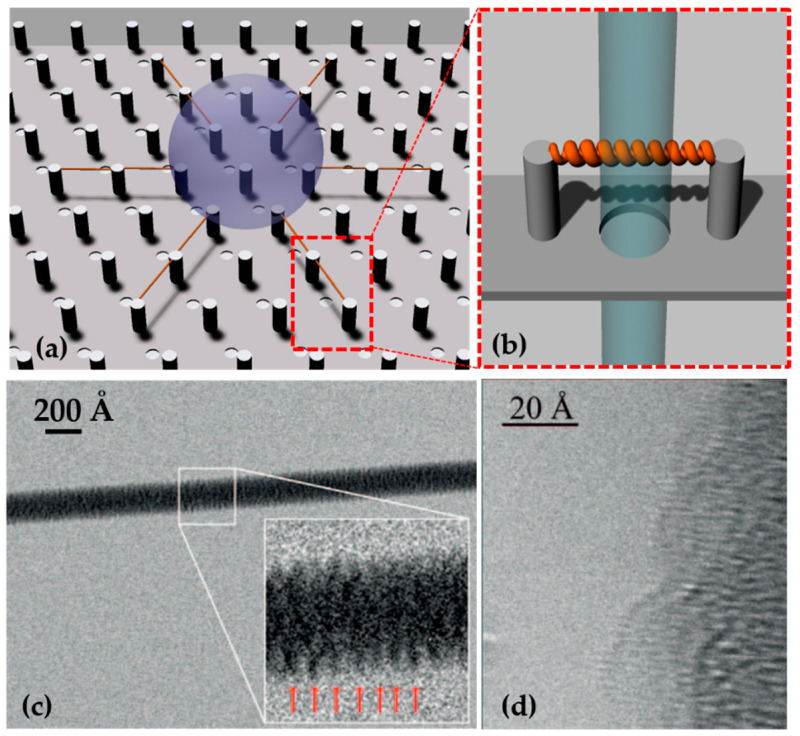
(**a**) A superhydrophobic device with holes between micropillars allows the dehydration of DNA droplet and the suspension of DNA fibers between micro-pillars. (**b**) This method permits the direct imaging of the suspended nucleic acid as the beam can freely pass through the hole. Adapted from [100] © 2017 with kind permission of Società Italiana di Fisica. (**c**) TEM image of an 80 Å DNA bundle suspended between micropillar, and in the inset, 10 periods of A-DNA. Reprinted with permission from [141]. © 2012 American Chemical Society. (**d**) A-DNA phase-contrast direct image acquired by HRTEM. The single helix is bound to a 100 Å DNA bundle. Reprinted with permission from [62] © 2015, The Authors.

Superhydrophobic devices, exploited in Section 3.1, were used for DNA suspension and imaging with TEM and HRTEM. Briefly, a droplet of a solution containing DNA and physiological buffer was pipetted on a superhydrophobic device with a pattern of silicon micropillars tailored to maximize the following dehydration. Water evaporation reduces the volume of the droplet, which moves from one pillar to the next in line; the nucleic acids diluted in the solution follow the same movement. At the end of the dehydration, an ordered grid of DNA molecules is suspended between micro-pillars and over the holes of the device. Upon exposure to the beam, it can be hit by the electrons and directly imaged without the need for contrast agents and becomes background-free (Figure 4). A thorough overview of the application of this method is detailed in [100]. The system has been tested for lambda double-strand DNA and calf thymus ssDNA, with a sequence length of ~50 kb [140]. In the first step, an unstained and background-free DNA bundle of 8 nm, located over the hole of the device was directly imaged by TEM working at an electron beam energy of 120 keV, revealing a periodicity of 2.7 *±* 0.2 nm, as reported for A-DNA fibers [141]. Recently, Marini et al. [142] introduced a circular pattern for the pillars to maximize dehydration efficiency, and their inter-distance was reduced to 12 μm to ideally cover the pitch with only one DNA helix. The DNA solution was finely tuned and the resulting free-standing DNA molecules were imaged using a spherical aberration (Cs) corrected HRTEM Titan 60-300 (ThermoFisher Scientific, Waltham, MA, USA) working at 80 keV. The results of imaging allowed the researchers to visualize the single base. In addition to the major and minor groove, diameter, tilt of the bases, and pitch of the helix already measured with other techniques, this approach made visible the features that were only previously available through modelling. In fact, the bases and the phosphate backbone were directly measured with sizes of 3.8 Å and 5.3 Å, respectively.

At the time of writing, the search for a single, free-standing DNA double helix is still ongoing as it cannot withstand beam damage. DNA fibers with lengths comparable to, or higher than, the pillar-pillar distance can be suspended and optimized. The ideal substrates are long DNA molecules, such as genomic DNA or RNA, rather than shorter sequences.

The mechanical properties of lambda dsDNA bundles were explored by Stassi et al. [143] with a different approach. DNA molecules were suspended between superhydrophobic silicon micropillars, obtaining the beam of a nano-bridge DNA resonator. They used four types of DNA molecules: Pristine dsDNA; lambda-dsDNA intercalated with the commercial dye YOYO-1; lambda-dsDNA intercalated with the commercial dye GelRed), and; DNA, which had interacted with a chemotherapeutic agent (Cisplatin). The flexural modes of the resulting nano-resonators were analyzed by Laser Doppler Vibrometer. The resonance frequency of a resonator depends not only on its geometry and mass, but also on the stiffness of the material of the beam. Therefore, the authors correlated the first mode resonance frequencies of the suspended structures to their effective Young’s moduli, demonstrating that DNA becomes stiffer when it interacts with bisintercalants, which clamps two bases each four nucleotides, while the chemotherapeutic agent disrupts H-bonds between the bases and softens the molecule. Different concentrations of CisPt were studied showing that, for a low concentration, the effective Young’s modulus of the molecule is closer to pristine DNA, while for the oversaturating concentration, a plateau is reached and the bundles are not affected proportionally by the addition of more chemotherapeutic agent. This suggests the possibility of evaluating the molecular dosage that is effectively bound to DNA molecules, opening several possible bio-medical applications.

## 7. Conclusions and Future Trends

In the last few decades, extensive research has been done to develop our understanding of the DNA structure, and to correlate morphology and composition with function. This review aims to point out recent technological advances and elucidate their corresponding achievements. Although a lot is known about DNA structure and detection, less is known about its fine details. The growing number of biological questions increases the need for new spatially and temporally resolved multidisciplinary approaches. The huge technical achievements of recent years will improve our understand and increase future research activities. Moreover, environmental fluctuations and stresses deeply alter nucleic acids behavior, arrangement, and the ability to interact with other molecules. To study such complex connections, interdisciplinary research is undoubtedly the only possible approach forward. Future biological questions, technical improvements, and implementation will increase growth in this field with the integration of spectroscopic approaches with, e.g., big bang tomography [187,188] and fast scanning AFM [94].

## Figures and Tables

**Table 1:** Summary of the approaches reported in this review with their application to DNA studies and the related references.

Approaches		Description	References
Microfluidic Devices	Labelled-based	Dot-blot for indirect relative concentrations evaluation	[57]
		DNA microarray devices for detection	[58,59,60,61,62,63]
	Label-free	Optical detection	[64,65]
		Electrochemical detection	[66,67,68,69,70,71,72]
	High resolution melting analysis	Structural studies	[73,74]
Microscopic approaches	Transmission Electron Microscopy	Graphene as ultrathin support for imaging	[75,76,77]
		Cryogenic Electron Microscopy for imaging	[78,79,80]
	Atomic Force Microscopy	Imaging, force spectroscopies, and mechanical studies	[81,82,83,84,85,86,87,88,89,90,91,92,93,94]
Spectroscopic approaches	Raman and Fourier Transform Infrared spectroscopy	Raman spectroscopy and Super Hydrophobic Devices for conformational analysis	[95,96,97,98,99,100,101]
		Surface Enhanced Raman Spectroscopy for detection and non-conventional structures study	[102,103,104,105,106,107,108,109,110,111,112,113,114,115,116,117,118,119,120,121,122,123,124,125,126,127,128,129]
		Tip Enhanced Raman Spectroscopy for structural characterization	[130,131,132,133,134]
		Infrared spectroscopy for the study of structure and dynamics of genomic or synthetic DNA	[135,136]
	Vibrometric analysis	Analysis in static and dynamic mode of DNA mechanical properties	[137,138]
Unconventional approaches	Microfluidic devices and DNA Nanotechnology	Microfluidic device and DNA-Origami for plasmonic enantiomer separation	[139]
	Super Hydrophobic Devices and suspended DNA	DNA direct imaging at sub-nanometric resolution	[140,141,142]
		DNA nanomechanical analysis	[143]

## Data Availability

Not applicable.
